# Understanding coronavirus disease (COVID-19) risk perceptions among the public to enhance risk communication efforts: a practical approach for outbreaks, Finland, February 2020

**DOI:** 10.2807/1560-7917.ES.2020.25.13.2000317

**Published:** 2020-04-02

**Authors:** Anna-Leena Lohiniva, Jussi Sane, Katja Sibenberg, Taneli Puumalainen, Mika Salminen

**Affiliations:** 1 Finnish Institute for Health and Welfare, Helsinki, Finland

**Keywords:** risk perception, risk communication, pandemic preparedness and response, Finland, outbreaks

## Abstract

Understanding risk perceptions of the public is critical for risk communication. In February 2020, the Finnish Institute for Health and Welfare started collecting weekly qualitative data on coronavirus disease (COVID-19) risk perception that informs risk communication efforts. The process is based on thematic analysis of emails and social media messages from the public and identifies factors linked to appraisal of risk magnitude, which are developed into risk communication recommendations together with health and communication experts.

In February 2020, The Finnish Institute for Health and Welfare (THL) initiated a practical exercise to analyse risk perceptions and trust towards public authorities in the context of coronavirus disease (COVID-19). The process allows qualitative data collection and analysis in real time, based on social media posts and emails from the public. Its objective is to inform risk communication efforts on weekly basis. This process was developed in collaboration with a medical anthropologist and experts in risk communication and public health. The information can be used to help identify appropriate responses and communication strategies to COVID-19 related topics of public interest. This paper describes the methodology and the results of the first 3 weeks of the exercise.

## Exploring COVID-19 subjective risk perceptions 

This approach uses qualitative data collection and analysis methods to explore the subjective COVID-19 risk perceptions of members of the public who request information from THL or comment on COVID-19 via THL’s email or social media. The process takes an anthropological approach to explore the meanings of risk and the elements that steer the assessment of a risk situation. It uses a conceptual framework of subjective perception of risk, reflecting individual, societal and cultural contexts [1]. It also includes exploring the perceived trust [2] towards those who are communicating about COVID-19 in Finland.

The narrative data from email accounts and social media channels are forwarded daily to a data analyst. Thematic analysis is carried out weekly to systematise and recognise emerging themes by organising the narrative data according to the risk model, identifying codes and categories within each domain, followed by developing concepts based on data interpretation [3]. The concepts are developed into operational recommendations through the methodology of knowledge co-production. This methodology uses the knowledge of different disciplines to ensure that findings and resulting recommendations are seen as relevant [4], following best practices for public health and risk communication [5]. The process requires expertise in qualitative analysis and a robust system for retrieving social media data from various sources. Qualitative software can facilitate organising the data.

## Risk perception concepts and risk communication recommendations 

The data presented in this paper is based on information collected during three consecutive weeks, between 3 and 25 February 2020. The narrative data was based on the 116 social media posts and emails from the public, and the findings related to five risk perception domains: catastrophic potential, probability of dying, reasons for exposure, the belief of being in control of the situation, and trust towards authorities (Table).

**Table:** Risk perception and risk communication analytical framework, based on data collected from social media posts and emails from the public on coronavirus disease, Finland, 3–25 February 2020 (n = 116)

Data	Concepts	Risk communication recommendations
Risk perception domain: catastrophic potential		
Strong wording describing the epidemic, catastrophe, worldwide threat	Emotional response	Avoid downplaying strong feelings
Beliefs that the epidemic is expanding tremendously	Anticipation of growth of the epidemic	Provide facts
Beliefs that the epidemic is growing because no actions have been taken by authorities	Belief that authorities lack interest in taking action	Express care and concern
Lack of personal protective equipment such as masks, lack of guaranteed places in health facilities, food, medicine, respirators	Suspicion that authorities lack the ability to take action	Share facts of what is known about available resources
Risk perception domain: probability of death		
A large number of people are likely to die; death is likely to persons who belong to risk groups	Death is uncontrollable, death is unpredictable	Emphasise known facts about mortality of COVID-19
Death is inevitable if no action is taken by authorities	Consequence of inaction by authorities	Emphasise actions taken by authorities
Risk perception domain: reasons for exposure		
Location of transmission: airports, and places with known confirmed cases; Mode of transmission: through contact with Chinese nationals, people coming from China, foreign patients and confirmed COVID-19 patients	Localised epidemic; stigmatising attitude towards foreign nationals	Humanise infected people by telling stories
Location of transmission: public transportation linked with airports and foreign passengers	Crowded places; stigmatising attitudes towards foreign nationals	Emphasise handwashing and cough etiquette as effective ways to prevent COVID-19
Mode of transmission: people who have resided in foreign countries	Epidemic can be anywhere; people linked with foreign countries	Emphasise known facts about global situation
Risk perception domain: belief of controllability		
Government can control the situation though restriction and making financial resources available	No perceived control over the epidemic	Emphasise handwashing and cough etiquette as effective ways to prevent COVID-19
Trust in the authorities		
Information content: information is hidden, differs from other countries, too optimistic	Unreliable information	Repeat information and provide an explanation (reason)
Actions of authorities are slow, no travel restrictions, no guaranteed places in health facilities, no isolation, no airport surveillance	Insufficient restrictions	Communicate actions

### Catastrophic potential

The analysis identified four concepts linked to catastrophic potential: ‘emotional response’, ‘anticipation of growth of the epidemic’, ‘belief that authorities are not interested in taking action’ and ‘suspicion that authorities are not able to take action’. Lack of knowledge generated further uncertainty that also increased perceptions of catastrophic potential. Accordingly, risk communication recommendations included avoiding downplaying strong emotions, providing factual information, expressing concern and care and sharing facts about available resources for pandemic planning.

### Probability of death

The analysis identified two different concepts increasing the perception of the probability of death during the COVID-19 epidemic. ‘Death’ was described as ‘uncontrollable’ and perceived as ‘likely’ since authorities were perceived as having taken insufficient actions to protect the public. Risk communication recommendations included emphasising known facts about case fatality ratios in different age groups and about actions taken by authorities.

### Reasons for exposure

Reasons for exposure to COVID-19 were believed to be ‘contact with infected persons’, ‘people coming from abroad’ or ‘foreign nationals’. The concepts linked with types of exposures included ‘stigmatising attitudes towards foreign nationals’ and ‘individuals who have resided in or travelled to foreign countries’. Communication recommendations included humanising individuals with COVID-19 by personalising a risk story that generates empathy among the public.

### Controllability beliefs

The findings showed that there was a lack of the belief that a person can individually control the spread of the epidemic and instead there was a strong belief that authorities can do that. Risk communication recommendations included emphasising what individuals can do to avoid spreading the infection such as hand hygiene, cough etiquette and avoiding touching eyes, nose and mouth.

### Trust

The analysis identified that trust in authorities was discussed through ‘distrust of information provided’ and ‘actions taken by the authorities’. Risk communication recommendations included repeating and explaining information given earlier to the public, and communicating the actions taken.

## Discussion

This qualitative data collection provided evidence-based recommendations for risk communication purposes. It proved to be a practical and participatory way to develop messages that can be used during the epidemic. This is critical as risk communication programmes must produce trustworthy and relevant information during emergencies to inform people about risk, influence behavioural change, and encourage participation in decision making about emergency measures. Communication must be meaningful and understood by those receiving it [6-9]. 

Knowing which risk perceptions influence a complex phenomenon, affected by multiple psychological, societal and cultural factors that are changing in place and time [1,10-12], is central for pandemic preparedness and planning. These risk perceptions guide individuals’ judgments and evaluations of threats, and can limit public compliance with and response to information communicated by authorities. Risk communication should be based on a sound understanding of the factors underlying risk perception, risk attitudes and trust towards communicating authorities [5,12,13]. 

This exercise focused on context specific explanations of risk in which the subjective meaning of risk is central. Accordingly, qualitative methods were considered appropriate to understand and explore the meaning behind risk perceptions. In the future, these perceptions could also be quantified through scaling and survey techniques to assess, for example, if trust towards authorities is growing or decreasing. 

People who contact health authorities during an emergency are often highly emotional and have strong opinions [9]. Therefore, they do not represent the risk perceptions of the general public in Finland. The strengths of this exercise are its ability to produce culturally competent and context-specific risk communication messages that are readily available for risk communicators during the COVID-19 response. 
